# The Association between Warning Label Requirements and Cigarette Smoking Prevalence by Education-Findings from the Global Adult Tobacco Survey (GATS)

**DOI:** 10.3390/ijerph14010098

**Published:** 2017-01-21

**Authors:** Ce Shang, Jidong Huang, Kai-Wen Cheng, Yanyun He, Frank J. Chaloupka

**Affiliations:** 1 Institute for Health Research and Policy, University of Illinois at Chicago, Chicago, IL 60608, USA; kaiwenc19@gmail.com (K.-W.C.); fjc@uic.edu (F.J.C.); 2 School of Public Health, Georgia State University, Atlanta, GA 30303, USA; jhuang17@gsu.edu; 3 Department of Economics, University of Illinois at Chicago, Chicago, IL 60607, USA; heyanyun1972@hotmail.com

**Keywords:** cigarette warning labels, smoking prevalence, disparity, education, GATS

## Abstract

Introduction: The Guidelines for the implementation of Article 11 of the World Health Organization (WHO) Framework Convention on Tobacco Control (FCTC) require that cigarette health warning labels should include pictures and take up 50% or more of the principal display area. This study examined how the association between large pictorial warnings, those covering ≥50% of the front and back of the package, and the prevalence of cigarette smoking varies by educational attainment. Methods: We pooled individual-level tobacco use data from the Global Adult Tobacco Survey (GATS) in 18 countries between 2008 and 2013 and linked them with warning label requirements during the same period from the MPOWER database and reports regarding warnings. The respondents’ self-reported exposure to warnings was examined according to education. Logistic regressions were further employed to analyze education-specific associations between large pictorial warnings and smoking prevalence, and whether such association differed by education was examined using an interaction test. Results: At the time of the survey, eight out of 18 countries had imposed graphic warning labels that covered ≥50% of the package. These warnings were associated with a 10.0% (OR = 0.89; 95% CI: 0.81, 0.97; *p* ≤ 0.01) lower cigarette smoking prevalence among adults with less than a secondary education or no formal education, but not among respondents with at least a secondary education. Less educated respondents were also less likely to be exposed to warnings in all 18 countries. The association between strong warnings and lower smoking prevalence among less educated respondents could be greater if their exposure to warnings increases. Conclusions: Prominent pictorial warning labels can potentially reduce health disparities resulting from smoking across different education levels.

## 1. Introduction

There is a rich evidence base that supports warning labels being an effective tobacco control policy as they serve as a powerful intervention providing health information to both smokers and nonsmokers [1,2,3,4,5], and increase knowledge of the harmful effects of smoking [5,6]. An increasing number of studies further illustrate that graphic warnings are more effective in reducing smoking than text-only warnings [7,8]. Graphic warning labels have been shown to reduce smoking prevalence and increase attempts to quit in Canada, which has adopted prominent graphic warning labels covering at least 50% of the front and back of the package since 2001 [9,10,11]. Growing evidence also shows that large prominent warnings affect a variety of smoking-related outcomes in other high-income countries (HICs), as well as in many low- and middle- income countries (LMICs) [12,13,14,15,16,17].

Globally, health warning labels have been increasingly implemented in many countries in the past decade. In 2005, the first World Health Organization (WHO) treaty—the Framework Convention on Tobacco Control (FCTC)—entered into force, and with 180 signing parties as of 2016, it became one of the most rapidly ratified treaties in the United Nation’s history [18]. The guidelines for the implementation of Article 11 of the WHO FCTC, the article addressing warning labels, produced in 2008, recommended that Parties adopt pictorial labels on packages that cover at least 50% of the principal display area [19]. These guidelines also provided recommendations for other characteristics that warnings should have, such as their location, color, rotation, content, language, and source attribution. The WHO also includes warnings as one of the most effective and cost-effective tobacco control measures including in its MPOWER package [20]. As of 2016, 105 countries or jurisdictions required pictorial warnings, with 77 countries or jurisdictions implementing such warnings [21]. Countries or jurisdictions that required large warnings taking up at least 50% of the front and back packages had also grown from 24 in 2008 to 94 in 2016 [21].

Despite strong evidence on the effectiveness of warning labels in influencing smoking-related outcomes [12,13,14,15,16,17], few studies have investigated differences in the effectiveness of warnings by socioeconomic status (SES), especially in LMICs. One review study concluded that health warnings had a neutral-to-positive equity impact, and that the responsiveness to warnings was either similar across SES groups or stronger among lower SES than among higher SES [22]. However, many studies cited in the review only investigated text-only warnings and the evidence exclusively came from HICs [17,22,23].

Several studies suggest that education may play an important role in the responsiveness to warning labels. Hitchman et al. [17] found that the effectiveness of the European Union text-only warnings was stronger among less educated populations in France, Germany, the Netherlands, and the United Kingdom. In an experimental setting, Cantrell [23] compared the impacts of graphic warnings on reactions to warning labels by SES groups and found that those with a high school education or less may be more responsive to warning labels in the United States than those with more than a high school education. Meanwhile, less educated smokers are also more likely to be offered smuggled cigarettes in some countries, rendering them less likely to be exposed to appropriate warning labels [24,25]. The combined evidence suggests that increased exposure to graphic warning labels can potentially reduce the health disparities caused by smoking.

Furthermore, compared to pictorial warnings in HICs, such warnings in LMICs may be more likely to have a positive equity impact on health. Since graphic warnings have been shown to be more effective than text-warnings [2], illiterate people in LMICs may benefit more from graphic warnings through better communications regarding health risks. Another study examined the perceived effectiveness of graphic warnings among various socioeconomic groups in Mexico and found that less educated, older, and female adults tended to consider such warnings to be more effective [26].

To add evidence on the differential impact of warning labels, this study examined the association between large pictorial warning labels and cigarette smoking prevalence by adults’ educational attainment, using data primarily from LMICs. We additionally explored factors that may influence the exposure to warning labels by educational attainment in these countries. 

## 2. Data and Methods

### 2.1. Data Sources

#### 2.1.1. Global Adult Tobacco Survey (GATS)

GATS is a nationally representative household cross-sectional survey of tobacco use among non-institutionalized adults aged 15 year or older. Conducted primarily in LMICs using comparable protocols and questionnaires, GATS was designed to assist countries in evaluating and forming effective tobacco control policies [27]. Specifically, all GATS countries used country-specific stratified multi-stage cluster sampling designs, and their surveys consisted of interviews with both a household screening and an individual component [27,28]. GATS surveys cover a variety of topics including tobacco use prevalence, economic factors, exposure to policies and marketing, etc., which allow for an in-depth analyses of key tobacco control measures while controlling for other confounders [29]. 

As of February 2016, 22 countries had released the data from their GATS surveys conducted between 2008 and 2013 on the Centers for Disease Control and Prevention website as part of Global Tobacco Surveillance System Data (GTSSData) [30]. These countries and their corresponding survey years and sample sizes are as follows: Argentina (2012; N = 6645), Bangladesh (2009; N = 9629), Brazil (2008; N = 39,425), China (2010; N = 13,354), Egypt (2009; N = 20,924), Greece (2013; N = 4359), India (2009; N = 69,269), Indonesia (2011; N = 8305), Malaysia (2011; N = 4250), Mexico (2009; N = 13,617), Nigeria (2012, N = 9765), Panama (2013; N = 16,962), the Philippines (2009; N = 9701), Poland (2009; N = 7840), Qatar (2013; N = 8571), Romania (2011; N = 4517), Russian Federation (2009; N = 11,406), Thailand (2009; N = 20,566), Turkey (2008; N = 9030), Ukraine (2010; N = 8518), Uruguay (2009; N = 5581), and Vietnam (2010; N = 10,383).

Among these 22 countries, Indonesia, Qatar, Bangladesh, and India were excluded because kretek, shisha or bidi smoking was also popular: kretek smoking was 31.5% in Indonesia, shisha smoking was 3.4% in Qatar, and bidi smoking was 9.2% in India and 11.2% in Bangladesh, respectively [30]. Cigarette smoking in these countries would also have been affected by marketing and regulation of these other products and thus pose a challenge to constructing comparable outcomes and predictors across countries. As a result, these four countries were dropped and 18 countries where cigarettes were the dominant smoked tobacco form were used in the analyses. The sample size of the combined 18 GATS surveys was 21,683.

Other than Argentina, the remaining 17 countries had all signed and ratified the WHO FCTC when GATS was conducted. At the time of the survey, there were 6 lower-middle-income countries (Egypt, Nigeria, The Philippines, Thailand, Ukraine, Vietnam), 10 upper-middle-income countries (Argentina, Brazil, China, Malaysia, Mexico, Panama, Romania, Russian Federation, Turkey, Uruguay), and two high-income countries (Greece, Poland). Among the 18 countries, there is one from the WHO’s African region (Nigeria), five from its American region (Argentina, Brazil, Mexico, Panama, Uruguay), one from its Eastern Mediterranean region (Egypt), six from the European region (Greece, Poland, Romania, Russian Federation, Turkey, Ukraine), one from the South-East Asian region (Thailand), and four from the Western Pacific region (China, Malaysia, the Philippines, Vietnam).

#### 2.1.2. MPOWER Scoring Package

Country-level warning label requirements and other tobacco control policies in the years 2008, 2010, 2012, and 2014 came from the WHO MPOWER database. The MPOWER package includes six key components: (M) monitoring tobacco use and prevention policies, (P) protecting people from tobacco smoke, (O) offering help to quit tobacco use, (W) warning about the dangers of tobacco, (E) enforcing bans on tobacco advertising, promotion and sponsorship, and (R) raising taxes on tobacco [4]. Other than the M score, which is a surveillance tool of tobacco epidemic, each POWER score encompasses a comprehensive set of policies that have been shown to reduce smoking and categorizes the degree of policy implementation into different levels. The levels of implementation were coded using scores from one to four or five, indicating the lowest to highest level of implementation [31]. 

### 2.2. Variables

#### 2.2.1. Outcome Variables 

All outcome variables came from the GATS from the 18 primarily cigarette using countries. The major outcome variable we examined was cigarette smoking prevalence, measured using standard questions that were common across GATS countries regarding cigarette consumption. More specifically, cigarette smoking prevalence was constructed using questions “On average, how many manufactured cigarettes do you currently smoke each day/each week?”, and “On average, how many hand-rolled cigarettes do you currently smoke each day/each week”. Cigarette smoking prevalence was constructed as a dichotomous variable that is coded as 0 for respondents who did not smoke any cigarettes, and as 1 for those who smoked either manufactured or hand-rolled cigarettes.

In addition to cigarette smoking prevalence, we also examined the respondents’ exposure to warnings as an intermediate outcome that would influence the association between warning labels and cigarette smoking. To assess the respondents’ exposure to warnings, we constructed a set of variables that are related to the chances that an individual saw the warning. Based on the aforementioned questions regarding the use of manufactured or hand-rolled cigarettes, we categorized cigarette use into exclusive use of manufactured cigarettes, exclusive use of hand-rolled cigarettes, and the use of both manufactured and hand-rolled cigarettes. As users of hand-rolled cigarettes may be less likely to see packages and warnings, this categorization illustrates the level of warning label exposure due to use patterns. 

Using the questions “In the last 30 days, did you see any phrases about the risks of smoking on cigarette packages?”, or “In the last 30 days, did you notice any health warnings on cigarette packages?”, we constructed measures for the exposure to warnings on packages. Answers to these questions were coded in three levels in GATS: did not see any cigarette packages, YES, and NO. Using these three levels, we constructed two dichotomous variables: “saw any cigarette packages” and conditional on seeing any packages, “whether individuals saw any warnings”. The first dichotomous variable measures exposure to packages and picks up differences in the availability of unpackaged cigarettes in the market, whereas the second variable measures respondents’ exposure to warnings once they see a package, which may reflect the influence of the size and visibility of warnings. If smuggled cigarettes existed, the second measure would also reflect the influence of illegal cigarettes that did not carry warnings or carried less prominent warnings. We further looked into the purchasing patterns of smokers and used the question “The last time you bought cigarettes for yourself, how many cigarettes did you buy?” to identify the prevalence of purchasing individual sticks or in forms other than packs or cartons that may prevent smokers from seeing warnings.

#### 2.2.2. Predictor Variables 

When estimating the association between warning labels and cigarette smoking prevalence, we controlled for a series of country- and individual- level confounders. In addition, common trends in tobacco use and the tobacco control environment across countries were controlled for using a linear year trend. 

Education and individual-level SES confounders were constructed using information from GATS. Education was obtained from the question “What is the highest level of education you have completed?” or similar questions. Despite varying education systems and structures across countries, the levels of highest education completed can be generally categorized into the following five groups: no formal schooling, primary, secondary, post-secondary, and college or higher. Details on how we coded this variable for each country can be found in Appendix A Table A2. Following the previous literature [32,33], we also constructed various control variables that are common across the 18 countries, including age in years, household size, and a dummy of being employed (see Appendix A Table A3). Additionally, according to the GATS economic analysis toolkit [34], a series of questions regarding assets, such as cell phone, television, refrigerator, car, etc., were used to construct a wealth index for each individual to control for the wealth effect on smoking behavior.

Information on cigarette warning labels was obtained from periodic international cigarette packaging and health warnings status reports, Euromonitor International cigarette and tobacco reports (http://www.euromonitor.com/), ERC reports (http://www.marketresearch.com/ERC-Statistics-Intl-plc-v1068/), the Tobacco Labeling Resource Centre website (http://www.tobaccolabels.ca/countries/canada/), and the MPOWER database [21,31] Based on the effective dates of warnings and their requirements concerning pictograms and size, for each country and year, we constructed a dichotomous variable for large pictorial warnings covering at least 50% of the front and back of package, with those that met these criteria coded as 1 and those that did not meet these criteria coded as 0.

In Appendix A Table A1, we present detailed information about warnings at the time of the survey in the 18 countries, including whether it was pictorial, the size of warnings on the front-, the back- and the combined front and back of the package, and whether misleading terms were banned. When the GATS was conducted, eight countries (Argentina, Brazil, Egypt, Malaysia, Panama, Thailand, Ukraine, and Uruguay) had large pictorial warnings covering ≥50% of the front and back of the package, whereas six countries (Vietnam, Turkey, Russian Federation, Poland, Greece, and China) imposed medium size text-only warnings covering 30%–49% of the pack, one country, (Romania) imposed a medium size pictorial warning of 35%, and three countries (Mexico, Nigeria, and the Philippines) had small text-only warnings covering <30% of the pack or unspecified sized text warnings. 

The MPOWER database also documents other characteristics of the warnings. The W score reflects the size of warning (average percent of the front and back of the package) and seven other characteristics: specific health warnings mandated; appear on individual packages as well as on any outside packaging and labelling used in retail sale; describe specific harmful effects of tobacco use on health; are large, clear, visible and legible (e.g., specific colors and font style and sizes are mandated); rotate; include pictures or pictograms; and are written in (all) the principal language(s) of the country [31]. GATS countries with large pictorial warnings also had reached the highest strength measured by the W score. That is, large warnings covering on average at least 50% of the front and back of the package with all seven recommended characteristics. In addition, these countries also had banned misleading terms in warnings (Appendix A Table A1) and thus met the WHO FCTC Article 11 guidelines regarding warning labels. 

Four of the six scores in the MPOWER package—P, O, E, and R—were included in the analyses as covariates in order to control for the tobacco control environment other than warning labels. The M score was not included because it measures surveillance of tobacco use rather than policies in a country. Because GATS was conducted in different calendar years between 2008 and 2013 and MPOWER data were biennial, POER scores in 2009, 2011, and 2013 were filled in using scores measured in 2008, 2010, and 2012, respectively. 

In addition to the tobacco control environment, we also controlled for country characteristics that are potentially highly correlated with smoking rates and the implementation of large graphic warnings, which are each country's stage of the tobacco epidemic and the literacy rate among adults age 15 or older. The stage of the tobacco epidemic was constructed for the survey year based on a model developed by Lopez, with values 1–4 representing the least to the most advanced stage [35]. This measure controls for predetermined factors that influence the difference in smoking prevalence across countries such as the trajectories of cigarette smoking due to tobacco use histories and shifts in demographics. Literacy rates among adults were obtained from the World Bank database, and because they were documented periodically, estimates for the nearest year when such information was available were matched to GATS data. As countries with lower literacy rates may have incentives to implement large graphic warnings while having a higher smoking rate, this variable helps to account for the potential reverse causality between warning label policies and smoking prevalence. This variable also controls for disparities in education across countries. 

### 2.3. Methods

Given that previous studies showed a significant difference in the exposure to warning labels by educational attainment in some countries [29,30], we started by comparing measures that are related to warning label exposure by individuals’ education levels. Specifically, we used the Chi-square value from a two-sample test to examine by education the differences among a set of variables that we consider to illustrate the degree of exposure, including “exclusive use of manufactured cigarettes”, “exclusive use of hand-rolled cigarettes”, “use of both manufactured and hand-rolled cigarettes”, “saw any cigarette packages” and, conditional on seeing any packages, “whether individuals saw any warnings”. As smokers may engage in tax avoidance or purchase illicit cigarettes [24], we further examined among smokers how “saw any cigarette packages”, “whether individuals saw any warnings”, and “whether the last purchase was in a form other than packs or cartons”, vary by education levels. 

Logistic regressions were used to analyze the association between warning label policies and cigarette smoking prevalence. Individual observations from the 18 country surveys were first stacked and then matched with POER scores and the dummy for large pictorial warnings using survey year and country identifiers. Analyses were performed first using the full sample and then for samples stratified by whether respondents had at least a secondary education. All regressions controlled for: country-level confounders including the stage of the tobacco epidemic, literacy rates, and POER scores that measure tobacco control environment; individual-level characteristics including gender, age in years, age squared, household size, employed dummy, and wealth index; and a linear year trend. When analyzing the full sample, regressions included an additional control variable reflecting whether the respondent had a less than secondary education or no formal education. Finally, to test the difference in the association between warning requirements and cigarette smoking prevalence by education, an interaction test was performed by analyzing a model with interaction terms of all other predictable variables and the education dummy. A significant estimate for the interaction term between warning requirements and the education dummy would indicate that their association varies by educational attainment. Throughout the analyses, the regressions were conducted using Stata Version 14.1. Estimates were evaluated using both odds ratios and the percent of change in smoking prevalence associated with large pictorial warnings.

## 3. Results

In Table 1, we present the summary statistics of the analytical samples. As described in the Data section, the adult sample consists of 18 primarily cigarette using countries from all WHO regions (Africa 1; Americas 5; East Mediterranean 1; Europe 6; Southeast Asia 1; Western Pacific 4). 

Other than Argentina, countries had all ratified the WHO FCTC and were obligated to eventually adopt the recommended policies. In the sample, the number of lower-middle-, upper-middle-, and high- income countries, is 6, 10, 2, respectively. After dropping observations with missing education, employment status, and wealth index, the sample size was 215,655. The mean (%) statistics show that over half (56.7%) of the sample at the time of survey lived in a country (Argentina, Brazil, Malaysia, Panama, Thailand, Ukraine or Uruguay) with a warning label that meets the WHO FCTC guidelines by requiring pictorial warnings covering at least 50% of the front and back of the cigarette package. The average POER scores suggest that countries in our analytical samples had implemented tobacco control policies but not at the highest level as reported in the MPOWER database. In these countries, the tobacco epidemic was at a relatively advanced stage, on average, and the average adult literacy rate was 90.4%. The sample characteristics were as follows: the percentage of males was 47.7%; the average age was 42.7 years; average household size was 3.7; the employment rate was 59.6%; and the wealth index was 0.68, suggesting that on average the respondent owned about 68% of the items listed as assets in the survey. The outcome we investigated, cigarette smoking prevalence among adults, was on average 20.8%, whereas the population with less than a secondary education was 40.8%. 

Table 2 contains two-sample test or Chi-square test results showing degrees of exposure to warning labels by education and by country. These results show that less educated populations and less educated smokers were less likely to be exposed to warning labels than their more educated counterparts. Specifically, less educated people were more likely to exclusively use hand-rolled cigarettes (in China, Egypt, Malaysia, the Philippines, Poland, Russian Federation, Thailand, Turkey, Ukraine, Uruguay, and Vietnam), or to use both manufactured and hand-rolled cigarettes (in Brazil, China, the Philippines, Poland, Russian Federation, Ukraine, Uruguay, and Vietnam). Alternatively, less educated people were less likely to exclusively use manufactured cigarettes (in Argentina, Brazil, China, Greece, Malaysia, Mexico, Poland, Romania, Russian Federation, Thailand, Turkey, Ukraine, and Uruguay) and thus may be less likely to be exposed to warnings on the package. Less educated people were also less likely to see packages in the last month (in countries other than the Philippines), or notice warnings when they saw packages (in all 18 countries). 

Less educated smokers were more likely to purchase in forms other than packs or cartons (e.g., in sticks, or did not purchase) in the last month (in Brazil, China, Egypt, Panama, Poland, Russian Federation, and Romania), were less likely to see packages in the last month (in Brazil, China, Malaysia, Mexico, Nigeria, the Philippines, Russian Federation, Thailand, Ukraine, and Vietnam), and were less likely to notice warnings when they saw packages (in countries other than Argentina, Egypt, Greece, and Poland). In conclusion, a common pattern emerges that less educated people and less educated smokers were less likely to be exposed to warning labels than their more educated counterparts.

In Table 3, we present logistic regression results for the association between large pictorial warning labels and cigarette smoking prevalence, analyzed using all adults (column 1) and samples stratified by educational attainment (columns 2 and 3). We found that, when pooling both education levels, large pictorial warning labels that cover ≥50% of the front and back of the package were marginally associated with a 2.3% lower cigarette smoking prevalence at a 10% level (*p* ≤ 0.1). In addition, large pictorial warning labels were associated with a 10.0% lower cigarette smoking prevalence among adults with less than a secondary education (*p* ≤ 0.01), but the association was not statistically significant among adults with at least a secondary education. Interaction test results further confirms that this association significantly differed by education at a 0.1% level (*p* ≤ 0.001). When pooling both education levels, large pictorial warning labels were marginally associated with a 3.0% lower cigarette smoking prevalence at a 10% level (*p* ≤ 0.1). 

## 4. Discussion

Using GATS data from 18 primarily LMICs, we analyzed how the association between large pictorial warnings and cigarette smoking prevalence differs by educational attainment, measured by whether a respondent had at least a secondary education or not. We found that large pictorial warnings were marginally associated (*p* ≤ 0.1) with a 2.3% lower smoking prevalence among all adults. When stratified by education, such large warnings were only significantly associated (*p* ≤ 0.01) with a 10.0% lower smoking prevalence among adults with less than a secondary education, and not among those with a secondary education or higher. The interaction test further illustrates that the association between large pictorial warnings and cigarette smoking significantly differs (*p* ≤ 0.001) by education. We further found that the exposure to warning labels was also significantly lower among less educated smokers than among more educated smokers in all 18 countries. These results suggest that the effectiveness of graphic warning labels among less educated population could be even stronger if their exposure to warning labels could be increased.

These findings are consistent with findings from previous studies that concluded that less educated people were more responsive to graphic warning labels or perceived graphic warning labels as more effective [17,22,23,26]. The combined evidence suggests that prominent graphic warnings may promote health equity by reducing smoking disparities across populations with different educational attainment. 

In addition, as all 18 GATS countries required some warnings on cigarette packages, results also indicate that prominent pictorial warnings, compared with text-only warnings and small pictorial warnings that cover less than 50% of packages, were associated with lower cigarette smoking prevalence among adults with less than a secondary education. These findings are in line with previous studies that found pictorial warnings to be more effective in reducing smoking than text-only warnings [3,4].

Furthermore, when large pictorial warnings were implemented in GATS countries, these warnings also met the WHO FCTC guidelines about other warning characteristics (see Appendix A Table A1). As a result, our results can also be interpreted as findings pertaining to health warnings that meet the WHO FCTC guidelines regarding size, pictograms, rotation, language, etc. and warnings that were implemented at the highest level measured in MPOWER package. 

This study is the first to examine how warning labels were differentially associated with smoking prevalence by education in primarily LMICs. Compared with HICs where the literacy rate was almost 100%, 15 out of the 16 GATS LMICs in the analyses had a literacy rate lower than 100%. Because illiterate people cannot read text-only warning labels and large pictorial warnings were found to be associated with lower smoking prevalence among less educated population, our findings provide some supports that illiterate populations may benefit more from graphic warning labels through a better understanding of the risks of smoking via pictograms. 

The findings of this study are also very important because many high tobacco using countries are LMICs that currently do require large pictorial warnings [21]. For example, China has the largest burden related to smoking yet it only implements text-only warnings that occupy 35% of the package. The Russian Federation, another country where cigarette smoking prevalence is high, currently adopts graphic warnings that cover 40% of the package [21]. The implementation of large pictorial warnings in these countries could potentially reduce smoking prevalence in the less educated population. Moreover, as studies also show that less educated people are also more likely to be smokers in these countries [32,36,37], large pictorial warning labels may also reduce health disparities across educational levels in these high tobacco using countries.

This study is subject to a few limitations. First, the pooled GATS data were in a one-timecross-sectional format that do not allow us to identify the causal impact of warning labels in reducing smoking prevalence [38,39] Second, due to the limited number of countries and years in the pooled data, standard errors were not clustered at either country or year level and could be underestimated [40]. Third, we could not identify the exact sources of lowered exposure to warning labels among less educated adults, for example, whether it was because less educated respondents were more likely to buy illicit cigarettes or because they paid less attention to packages in certain countries. Fourth, GATS surveys were conducted primarily in LMICs; the health equity impact of pictorial warnings could differ in HICs. Last, we did not investigate warnings for other tobacco products that may also influence cigarette smoking prevalence. Future studies should address these limitations. 

## 5. Conclusions

This is the first study that utilizes regression methods to study the association between large pictorial warnings and cigarette smoking prevalence among adults by education in primarily LMICs. Our findings suggest that the association between prominent graphic warnings and lower smoking prevalence was greater among less educated adults and can potentially become even stronger if less educated adults are equally exposed to warnings as more educated ones. Large pictorial warning labels may be an effective tool in reducing health disparities attributable to smoking across groups with different educational attainment. Our findings provide strong evidence to support the warning label guidelines for Article 11 of the WHO FCTC.

## Figures and Tables

**Table 1:** Summary Statistics, all observations and by More versus Less education.

Variables	All	More Education	Less Education
	Mean/% (S.D.)	Mean/% (S.D.)	Mean/% (S.D.)
*Country-level variables*			
Pictorial Warning Labels ≥ 50%	56.70% (49.54%)	57.15% (49.49%)	56.05% (49.63%)
P score	2.78 (1.09)	2.67 (1.01)	2.95 (1.18)
O score	4.07 (0.74)	4.07 (0.76)	4.06 (0.70)
E score	3.53 (0.98)	3.56 (0.90)	3.49 (1.08)
R score	3.78 (0.64)	3.81 (0.66)	3.73 (0.62)
Stage of epidemic	2.86 (0.95)	2.92 (0.92)	2.79 (0.99)
Literacy rate	90.39% (11.31%)	91.08% (10.84%)	89.39% (11.89)
*Individual-level variables*			
Cigarette smoking	20.75% (40.55%)	22.18% (41.55%)	18.69% (38.98%)
Male	47.71% (49.95%)	49.98% (50.00%)	44.42% (49.69%)
Employed	59.60% (49.04%)	65.31% (47.58%)	51.33% (49.95%)
Household size	3.74 (2.19)	3.61 (1.91)	3.92 (2.53)
Age	42.72 (17.39)	38.08 (14.94)	49.45 (18.45)
Wealth index	0.68 (0.24)	0.76 (0.20)	0.58 (0.26)
<Secondary education	40.84% (49.15%)	--	--
N	215,655	127,581	88,074

Note: the distribution of education by category is as the following: no formal education or less than primary 19.27% (39.44%), primary education 21.57% (41.12%), secondary education 40.55% (49.10%), post-secondary education 4.80% (21.37%), and college or higher 11.49% (31.89%).

**Table 2:** Measures Related to Exposure to Warning Labels by Education Levels by Country (N = number of observations in the sample).

	**Argentina**		**Brazil**		**China**		**Egypt**		**Greece**	
**Education**	**More**	**Less**	**More**	**Less**	**More**	**Less**	**More**	**Less**	**More**	**Less**
Cigarette use pattern and exposure to warnings among general population										
Exclusively Hand-rolled	0.07% (N = 4575)	0.10% † (N = 1980)	--	--	0.65% (N = 7493)	4.03% (N = 5856)	0.00% (N = 11,697)	1.19% (N = 9221)	15.63% (N = 3122)	1.38% (N = 1235)
Exclusively Manufactured	25.44% (N = 4575)	20.96% (N = 1980)	12.19% (N = 30,485)	10.48% (N = 8940)	28.80% (N = 7493)	20.41% (N = 5856)	16.15% (N = 11,697)	16.82% † (N = 9221)	28.57% (N = 3122)	17.00% (N = 1235)
Hand-rolled and Manufactured	0.61% (N = 4575)	0.30% † (N = 1980)	2.84% (N = 30,485)	10.76% (N = 8940)	1.20% (N = 7493)	2.60% (N = 5856)	0.05% (N = 11,697)	0.05% † (N = 9221)	1.38% (N = 3122)	0.24% (N = 1235)
Saw packages in the past month	79.26% (N = 4575)	72.93% (N = 1980)	88.63% (N = 30,485)	79.19% (N = 8940)	88.86% (N = 7493)	82.92% (N = 5856)	96.22% (N = 11,697)	93.83% (N = 9221)	90.49% (N = 3122)	71.82% (N = 1235)
Saw warnings conditional on seeing packages in the past month	74.02% (N = 3626)	62.88% (N = 1444)	82.11% (N = 27,019)	67.15% (N = 7080)	75.59% (N = 6658)	43.27% (N = 4856)	98.19% (N = 11,255)	96.51% (N = 8652)	82.76% (N = 2825)	59.41% (N = 887)
Cigarette purchasing pattern and exposure to warnings among cigarette smokers										
Did not buy in packages or cartons during last purchase	10.23% (N = 1192)	8.79% † (N = 421)	36.98% (N = 4073)	42.00% (N = 1188)	4.03% (N = 2256)	7.30% (N = 1357)	3.27% (N = 1894)	7.21% (N = 1553)	10.63% (N = 988)	6.07% † (N = 214)
Saw packages in the past month	99.33% (N = 1195)	98.82% † (N = 423)	98.63% (N = 4582)	93.47% (N = 1899)	99.74% (N = 2297)	97.28% (N = 1583)	99.84% (N = 1895)	99.94% † (N = 1567)	98.95% (N = 1423)	99.57% † (N = 230)
Saw warnings conditional on seeing packages in the past month	85.76% (N = 1187)	83.97% † (N = 418)	93.56% (N = 4519)	82.20% (N = 1775)	93.63% (N = 2291)	70.45% (N = 1540)	98.84% (N = 1892)	98.66% † (N = 1566)	90.84% (N = 1408)	86.90% † (N = 229)
	**Malaysia**		**Mexico**		**Nigeria**		**Panama**		**The Philippines**	
**Education**	**More**	**Less**	**More**	**Less**	**More**	**Less**	**More**	**Less**	**More**	**Less**
Cigarette use pattern and exposure to warnings among general population										
Exclusively Hand-rolled	0.82% (N = 2185)	3.77% (N = 2043)	0.00% (N = 6182)	0.04% † (N = 7391)	0.04% (N = 4729)	0.08% † (N = 5024)	0.14% (N = 5178)	0.32% † (N = 11,727)	0.23% (N = 4758)	2.43% (N = 4942)
Exclusively Manufactured	17.48% (N = 2185)	13.71% (N = 2043)	14.88% (N = 6182)	10.81% (N = 7391)	3.13% (N = 4729)	2.97% † (N = 5024)	2.70% (N = 5178)	3.35% † (N = 11,727)	21.67% (N = 4758)	30.01% (N = 4942)
Hand-rolled and Manufactured	3.11% (N = 2185)	2.99% † (N = 2043)	0.31% (N = 6182)	0.19% † (N = 7391)	0.76% (N = 4729)	1.09% † (N = 5024)	0.75% (N = 5178)	0.91% † (N = 11,727)	0.11% (N = 4758)	1.36% (N = 4942)
Saw packages in the past month	88.15% (N = 2185)	78.17% (N = 2043)	84.57% (N = 6182)	77.38% (N = 7391)	78.35% (N = 4729)	75.28% (N = 5024)	94.61% (N = 5178)	91.86% (N = 11,727)	91.57% (N = 4758)	91.38% † (N = 4942)
Saw warnings conditional on seeing packages in the past month	89.25% (N = 1926)	83.28% (N = 1597)	65.24% (N = 5228)	46.81% (N = 5719)	42.19% (N = 3705)	21.81% (N = 3782)	64.36% (N = 4899)	48.93% (N = 10,772)	89.03% (N = 4357)	77.06% (N = 4516)
Cigarette purchasing pattern and exposure to warnings among cigarette smokers										
Did not buy in packages or cartons during last purchase	7.57% (N = 449)	11.24% † (N = 347)	41.85% (N = 939)	41.26% † (N = 812)	68.13% (N = 182)	74.26% † (N = 202)	43.33% (N = 180)	61.63% (N = 503)	75.29% (N = 1036)	64.51% (N = 1564)
Saw packages in the past month	99.57% (N = 468)	95.45% (N = 418)	98.83% (N = 939)	97.06% (N = 816)	100.00% (N = 186)	93.27% (N = 208)	97.31% (N = 186)	93.31% † (N = 538)	99.24% (N = 1047)	96.95% (N = 1670)
Saw warnings conditional on seeing packages in the past month	97.21% (N = 466)	88.22% (N = 399)	89.33% (N = 928)	76.26% (N = 792)	70.43% (N = 186)	48.97% (N = 194)	85.08% (N = 181)	70.72% (N = 502)	97.69% (N = 1039)	87.21% (N = 1619)
	**Poland**		**Romania**		**Russia**		**Thailand**		**Turkey**	
**Education**	**More**	**Less**	**More**	**Less**	**More**	**Less**	**More**	**Less**	**More**	**Less**
Cigarette use pattern and exposure to warnings among general population										
Exclusively Hand-rolled	1.30% (N = 6407)	2.56% (N = 1408)	0.23% (N = 3882)	0.00% † (N = 606)	0.03% (N = 10,035)	0.37% (N = 1367)	2.98% (N = 9367)	13.79% (N = 11,158)	0.95% (N = 2624)	1.67% (N = 6394)
Exclusively Manufactured	29.76% (N = 6407)	19.25% (N = 1408)	24.57% (N = 3882)	10.07% (N = 606)	41.90% (N = 10,035)	30.21% (N = 1367)	15.34% (N = 9367)	8.18% (N = 11,158)	38.22% (N = 2624)	22.46% (N = 6394)
Hand-rolled and Manufactured	1.00% (N = 6407)	1.99% (N = 1408)	0.46% (N = 3882)	0.33% † (N = 606)	0.45% (N = 10,035)	1.02% (N = 1367)	3.11% (N = 9367)	3.35% † (N = 11,158)	0.72% (N = 2624)	0.99% † (N = 6394)
Saw packages in the past month	84.81% (N = 6407)	78.91% (N = 1408)	86.40% (N = 3882)	65.35% (N = 606)	81.81% (N = 10,035)	71.18% (N = 1367)	95.09% (N = 9367)	89.34% (N = 11,158)	98.51% (N = 2624)	96.11% (N = 6394)
Saw warnings conditional on seeing packages in the past month	86.49% (N = 5434)	75.43% (N = 1111)	89.06% (N = 3354)	64.14% (N = 396)	84.40% (N = 8137)	74.48% (N = 960)	90.39% (N = 8907)	82.88% (N = 9968)	92.38% (N = 2585)	75.26% (N = 6145)
Cigarette purchasing pattern and exposure to warnings among cigarette smokers										
Did not buy in packages or cartons during last purchase	1.73% (N = 1966)	4.35% (N = 299)	6.80% (N = 971)	17.46% (N = 63)	1.72% (N = 4246)	3.52% (N = 426)	42.27% (N = 1765)	46.79% † (N = 1404)	4.31% (N = 1022)	6.64% † (N = 1505)
Saw packages in the past month	99.85% (N = 2054)	99.40% † (N = 335)	99.80% (N = 981)	98.41% † (N = 63)	99.95% (N = 4253)	99.54% (N = 432)	99.85% (N = 2007)	96.60% (N = 2826)	100.00% (N = 1047)	99.94% † (N = 1606)
Saw warnings conditional on seeing packages in the past month	96.73% (N = 2051)	94.89% † (N = 333)	98.16% (N = 979)	90.32% (N = 62)	95.95% (N = 4250)	92.33% (N = 430)	98.20% (N = 2004)	92.49% (N = 2730)	96.66% (N = 1047)	92.83% (N = 1605)
	**Ukraine**		**Uruguay**		**Vietnam**					
**Education**	**More**	**Less**	**More**	**Less**	**More**	**Less**				
Cigarette use pattern and exposure to warnings among general population										
Exclusively Hand-rolled	0.17% (7005)	0.88% (N = 1137)	1.57% (N = 2422)	8.33% (N = 3159)	0.09% (N = 5435)	1.09% (N = 4486)				
Exclusively Manufactured	30.46% (N = 7005)	11.79% (N = 1137)	19.82% (N = 2422	10.92% (N = 3159)	17.28% (N = 5435)	18.28% † (N = 4486)				
Hand-rolled and Manufactured	0.91% (N = 7005)	1.14% † (N = 1137)	2.64% (N = 2422)	5.48% (N = 3159)	0.09% (N = 5435)	0.80% (N = 4486)				
Saw packages in the past month	84.33% (N = 7005)	64.64% (N = 1137)	83.28% (N = 2422)	75.82% (N = 3159)	96.30% (N = 5435)	90.28% (N = 4486)				
Saw warnings conditional on seeing packages in the past month	79.97% (N = 5907)	53.06% (N = 735)	92.66% (N = 2017)	82.63% (N = 2395)	92.53% (N = 5234)	80.91% (N = 4050)				
Cigarette purchasing pattern and exposure to warnings among cigarette smokers										
Did not buy in packages or cartons during last purchase	5.70% (N = 2194)	8.22% † (N = 146)	33.58% (N = 545)	39.02% † (N = 533)	30.83% (N = 947)	31.87% † (N = 866)				
Saw packages in the past month	99.77% (N = 2210)	97.45% (N = 157)	99.66% (N = 582)	99.10% † (N = 781)	99.79% (N = 949)	98.45% (N = 905)				
Saw warnings conditional on seeing packages in the past month	96.92% (N = 2205)	87.58% (N = 153)	98.45% (N = 580)	94.32% (N = 774)	98.52% (N = 947)	93.15% (N = 891)				

Note: † denotes that the means were not significantly different by education at the 1% level.

**Table 3:** The Association between Large Pictorial Warning Labels and Cigarette Smoking Prevalence among Adults and By Education.

Independent Variables	All	More Educated	Less Educated
	(1)	(2)	(3)
Pictorial Warnings ≥ 50%	0.971 † (0.937, 1.006)	0.986 (0.945, 1.028)	0.885 ** (0.809, 0.967)
Less Educated (<secondary)	0.970 * (0.945, 0.996)	-	-
Percent Change in Smoking			
Pictorial Warnings ≥ 50%	−0.023 †	−0.011	−0.100 **
	(0.014)	(0.016)	(0.037)
N	215,655	127,581	88,074

Note: Odds ratio and corresponding 95% CI (in square brackets) are reported. Percent change in smoking was estimated using the formula log OR × (1 − mean prevalence) and corresponding standard errors (in parentheses) are reported in the lower panel of Table. Regressions also controlled for age, age squared, household size, employment status, wealth index, POER scores, the stage of tobacco epidemic, adult literacy rate, and a linear year trend. The interaction test shows that the association significantly differed by education levels, † *p* ≤ 0.1, * *p* ≤ 0.05, ** *p* ≤ 0.01.

## Appendix A

**Table A1:** Warning Label Requirements in the GATS survey years and in 2014.

Country	Pictorial ^a^		% Front		% Back		% Average		Misleading Terms Ban	
	GATS Year	2014	GATS Year	2014	GATS Year	2014	GATS Year	2014	GATS Year	2014
Argentina	Yes	Yes	50	50	50	50	50	50	Yes	Yes
Brazil	Yes	Yes	0	30	100	100	50	65	Yes	Yes
China	No	No	30	35	30	35	30	35	Yes	Yes
Egypt	Yes	Yes	50	50	50	50	50	50	Yes	Yes
Greece *	No	No	30	30	40	40	35	35	Yes	Yes
Malaysia	Yes	Yes	40	50	60	60	50	55	Yes	Yes
Mexico	No	Yes	0	30	50	100	25	65	Yes	Yes
Nigeria	No	No	Not specified	Not specified	Not specified	Not specified	Not specified	Not specified	No	Yes
Panama	Yes	Yes	50	50	50	50	50	50	Yes	Yes
The Philippines *	No	No	30	30	0	0	15	15	No	Yes
Poland *	No	No	30	30	40	40	35	35	Yes	Yes
Romania	Yes	Yes	30	43	40	53	35	48	Yes	Yes
Russia Federation	No	Yes	30	30	50	50	40	40	No	No
Thailand	Yes	Yes	50	85	50	85	50	85	Yes	Yes
Turkey	No	Yes	30	65	40	65	35	65	Yes	Yes
Ukraine	Yes	Yes	50	50	50	50	50	50	Yes	Yes
Uruguay	Yes	Yes	50	80	50	80	50	80	Yes	Yes
Vietnam	No	Yes	30	50	30	50	30	50	No	No

Note: ^a^ Countries with large pictorial warnings also met the 7 characteristics in W score. * In 2016, the Philippines, Greece, and Poland implemented pictorial warnings that are 50%, 65%, and 65% of the front and back of the package, respectively.

**Table A2:** Definition of education category by country.

Country	No Formal Schooling	Primary	Secondary	Post-Secondary, <College	College or Above
Argentina	Did not attend an educational institution, or kindergarten	Primary or E.G.B (Basic General Education)	Secondary, Polymodal	Tertiary not University	College/University, Post Graduate
Brazil	Do not know how to read or write, never attended school in the past, adult and youth literacy, Nursery, Class literacy-CA, Maternal, Kindergarten etc.	Elementary (primary)	Middle School (junior high, scientific, classical, etc.), regular EF or 1 degree, youth/adults or supplement elementary school or 1 degree, youth/adults or high school equivalent or 2nd degree	-	Top-graduation, Master’s or Ph.D.
China	No formal schooling, <primary school completed	Primary school completed, <secondary school completed	Secondary school completed, high school/technical secondary school	-	College/university completed, post-graduate degree completed
Egypt	No formal schooling; attended primary school, not completed	Primary school completed; attended preparatory school, not completed	Completed preparatory school, attended high school, not completed; completed high school/equivalent education, diploma	-	College/university completed, post-graduate degree completed.
Greece	No formal schooling, <primary school completed	Primary school completed, <secondary school completed	Secondary school completed, <high school completed, high school completed	-	College/university completed/technological educational institute, post graduate degree completed
Malaysia	No formal schooling, <primary school completed	Primary school completed, <secondary school completed	Secondary school completed, high school completed		College/university completed, post-graduate degree completed.
Mexico	No formal education	Primary	Secondary, Technical/TRADE Technical, Normal Basic, preparatory or vocational, technical high school		technical or trade degree, normal upper-level, Masters/Doctorate
Nigeria	No formal schooling, <primary school completed	Primary school completed	Junior or Senior secondary school completed	<college/university degree completed	College/university completed
Panama	No formal schooling, special education, <primary school completed	Primary school completed, <secondary school completed	Secondary school completed	Vocational, Superior no university	College/university completed, Post graduate degree completed
The Philippines	No formal education, elementary, not completed	Elementary completed, high school, not completed	High school completed	Post secondary year 1,2,3; college not completed	College completed, post graduate completed
Poland	No formal education, incomplete elementary	Elementary	Junior high school, vocational, secondary	Junior college	Bachelor’s degree, Master degree or higher
Romania	No formal education	Primary school completed	Secondary school completed, vocational, apprentice schools, high school completed	Post high school completed	College completed, university, post graduate degree completed
Russia	No formal education	Primary or some high school	High school	Vocational/trade school, some college	College, advanced degree
Thailand	No education/illiterate, <primary school	Primary school completed	Grade 6-gard 12/vocational education	Certificate/vocational education	≥Bachelor degree
Turkey	Not graduate	Elementary school/primary education	Secondary or Vocational secondary school, high school or equivalent	-	College or faculty, master/doctorate
Ukraine	No formal education	Primary school, <Secondary school completed	Basic or full secondary school completed, high school completed	-	College/university completed, post graduate degree completed
Uruguay	No formal schooling	Standard/special primary school	Basic cycle of high school/UTU/secondary bachelaurate/UTU technical bachelaurate/technical education/primary or secondary teaching degree	Tertiary, not university	University or similar, post-graduate
Vietnam	No formal education, not completed primary education	Completed primary education	Completed basic secondary/secondary education	Grad university/College/Specialized secondary education	College/university completed, post-graduated

**Table A3:** Variable Definitions.

Variable	Description
	*Country-level Policy variables*
Warning ≥ 50%	Indicator equals 1 if warning labels occupy at least 50% of the display area with all seven appropriate characteristic, 0 otherwise
POER scores	Four among the six MPOWER composite scores as ovriates
P score	Categorical variable: = 1 if data not reported or not categorized; = 2 if up to two public places completely smoke-free; = 3 if three to five public places completely smoke-free; = 4 if six to seven public places completely smoke-free; = 5 if all public places completely smoke-free (or at least 90% of the population covered by complete subnational smoke-free legislation; excluding pubs and bars where these are illegal)
O score	Categorical variable: = 1 if data not reported;= 2 if None; = 3 if there are NRT (Nicotine replacement therapy) and/or some cessation services (neither cost-covered); = 4 of there are NRT and/or some cessation services (at least one of which is cost-covered); = 5 if there are national quit line, and both NRT and some cessation services cost-covered
E score	Categorical variable: = 1 if data not reported; = 2 if complete absence of ban, or ban that does not cover national TV, radio and print media; = 3 if ban on national TV, radio and print media only; = 4 if ban on national TV, radio and print media as well as on some but not all other forms of direct and/or indirect advertising; = 5 if ban on all forms of direct and indirect advertising.
R score	Categorical variable: = 1 if data not reported; = 2 if ≤25% of retail price is tax; = 3 if 26%–50% of retail price is tax; = 4 if 51%–75% of retail price is tax; = 5 if >75% of retail price is tax
Stage of epidemic	Categorical variable that measures the stages of tobacco epidemic with levels 1–4 representing least to most advance stages.
Literacy rate	Percentage of literacy rates among adult population age 15 or older
	*Individual-level variables*
Cigarette smoking	Indicator equals 1 if the respondent smoked cigarettes in the past month, 0 otherwise
Male	Indicator equals 1 if male, 0 if female
Employment status	Indicator equal if being employed, 0 otherwise
Age	Age in years
Wealth index	The fraction of GATS-surveyed household items (electricity, flush toilet, and any other surveyed assets) that the respondents has in their possession
Household size	Number of household members
<secondary education	Binary indicators equals 1 if the completed education of a respondent is less than Secondary school or that the respondent never received formal schooling, 0 otherwise
	No formal education or <primary

## References

[1] Fong G.T., Hammond D., Hitchman S.C. (2009). The impact of pictures on the effectiveness of tobacco warnings. Bull. World Health Organ..

[2] Hammond D. (2011). Health warning messages on tobacco products: A review. Tob. Control.

[3] Hammond D., Fong G.T., McNeill A., Borland R., Cummings K.M. (2006). Effectiveness of cigarette warning labels in informing smokers about the risks of smoking: Findings from the International Tobacco Control (ITC) Four Country Survey. Tob. Control.

[4] Strahan E.J., White K., Fong G.T., Fabrigar L.R., Zanna M.P., Cameron R. (2002). Enhancing the effectiveness of tobacco package warning labels: A social psychological perspective. Tob. Control.

[5] World Health Organization (2011). WHO Report on the Global Tobacco Epidemic, 2011: Warning about the Dangers of Tobacco.

[6] Borland R., Wilson N., Fong G.T., Hammond D., Cummings K.M., Yong H.H., Hosking W., Hastings G., Thrasher J., Mcneill A. (2009). Impact of graphic and text warnings on cigarette packs: Findings from four countries over five years. Tob. Control.

[7] Noar S.M., Hall M.G., Francis D.B., Ribisl K.M., Pepper J.K., Brewer N.T. (2016). Pictorial cigarette pack warnings: A meta-analysis of experimental studies. Tob. Control.

[8] Thrasher J.F., Hammond D., Fong G.T., Arillo-Santillan E. (2007). Smokers’ reactions to cigarette package warnings with graphic imagery and with only text: A comparison between Mexico and Canada. Salud Publica Mex..

[9] Huang J., Chaloupka F.J., Fong G.T. (2014). Cigarette graphic warning labels and smoking prevalence in Canada: A critical examination and reformulation of the FDA regulatory impact analysis. Tob. Control.

[10] Azagba S., Sharaf M.F. (2013). The effect of graphic cigarette warning labels on smoking behavior: Evidence from the Canadian experience. Nicotine Tob. Res..

[11] Hammond D., Fong G.T., McDonald P.W., Cameron R., Brown K.S. (2003). Impact of the graphic Canadian warning labels on adult smoking behaviour. Tob. Control.

[12] Wilson N., Weerasekera D., Hoek J., Li J., Edwards R. (2010). Increased smoker recognition of a national quitline number following introduction of improved pack warnings: ITC Project New Zealand. Nicotine Tob. Res..

[13] Centers for Disease Control and Prevention (CDC) (2011). Cigarette package health warnings and interest in quitting—14 countries, 2008–2010. MMWR Morb. Mortal. Wkly. Rep..

[14] Thrasher J.F., Villalobos V., Szklo A., Fong G.T., Perez C., Sebrie E., Sansone N., Figueiredo V., Boado M., Arillo-Santillán E. (2010). Assessing the impact of cigarette package health warning labels: A cross-country comparison in Brazil, Uruguay and Mexico. Salud Publica Mex..

[15] Fathelrahman A.I., Omar M., Awang R., Borland R., Fong G.T., Hammond D., Zain Z. (2009). Smokers’ responses toward cigarette pack warning labels in predicting quit intention, stage of change, and self-efficacy. Nicotine Tob. Res..

[16] Fathelrahman A.I., Omar M., Awang R., Cummings K.M., Borland R., Bin Mohd Samin A.S. (2010). Impact of the new Malaysian cigarette pack warnings on smokers’ awareness of health risks and interest in quitting smoking. Int. J. Environ. Res. Public Health.

[17] Hitchman S.C., Mons U., Nagelhout G.E., Guignard R., McNeill A., Willemsen M.C., Driezen P., Wilquin J.L., Beck F., Du-Roscoat E. (2012). Effectiveness of the European Union text-only cigarette health warnings: Findings from four countries. Eur. J. Public Health.

[18] World Health Organization (2013). MPOWER in Action: Defeating the Global Tobacco Epidemic.

[19] World Health Organization (2008). Guidelines for Implementation of Article 11 of the WHO Framework Convention on Tobacco Control (Packaging and Labelling of Tobacco Products).

[20] World Health Organization (2008). MPOWER: Six Policies to Reverse the Tobacco Epidemic.

[21] Canadian Cancer Society (2016). Cigarette Package Health Warnings—International Status Report.

[22] Brown T., Platt S., Amos A. (2014). Equity impact of population-level interventions and policies to reduce smoking in adults: A systematic review. Drug Alcohol Depend..

[23] Cantrell J., Vallone D.M., Thrasher J.F., Nagler R.H., Feirman S.P., Muenz L.R., He D.Y., Viswanath K. (2013). Impact of tobacco-related health warning labels across socioeconomic, race and ethnic groups: Results from a randomized web-based experiment. PLoS ONE.

[24] Ross H. (2015). Understanding and Measuring Cigarette Tax Avoidance and Evasion: A Methodological Guide. http://tobacconomics.org/wp-content/uploads/2015/03/Ross_Methods_to_Measure_Illicit-Trade_03-17-15.pdf.

[25] Gruber J., Sen A., Stabile M. (2003). Estimating price elasticities when there is smuggling: The sensitivity of smoking to price in Canada. J. Health Econ..

[26] Hammond D., Thrasher J., Reid J.L., Driezen P., Boudreau C., Santillan E.A. (2012). Perceived effectiveness of pictorial health warnings among Mexican youth and adults: A population-level intervention to reduce tobacco related inequities. Cancer Causes Control.

[27] Global Adult Tobacco Survey Collaborative Group (2010). Global Adult Tobacco Survey (GATS): Sample Design Manual.

[28] Global Adult Tobacco Survey Collaborative Group (2010). Global Adult Tobacco Survey (GATS): Field Interviewer Manual.

[29] Global Adult Tobacco Survey Collaborative Group (2010). Global Adult Tobacco Survey (GATS): Core Questionnaire with Optional Questions.

[30] Asma S., Mackay J., Song S.Y., Zhao L., Morton J., Palipudi K.M., Bhatti L., Caixeta R.B., Chandora R., Dias R.C. (2015). The GATS Atlas.

[31] World Health Organization (2015). WHO Report on the Global Tobacco Epidemic, 2015: Raising Taxes on Tobacco Is the Fifth in a Series of WHO Reports that Tracks the Status of the Tobacco Epidemic and the Impact of Interventions Implemented to Stop It.

[32] Shang C., Chaloupka F., Kostova D. (2014). Who quits? An overview of quitters in low-and middle-income countries. Nicotine Tob. Res..

[33] Kostova D., Chaloupka F.J., Shang C. (2015). A duration analysis of the role of cigarette prices on smoking initiation and cessation in developing countries. Eur. J. Health Econ..

[34] World Health Organization (WHO), WHO Tobacco Free Initiative (WTFI) (2010). Economics of Tobacco Toolkit: Economic Analysis of Demand Using Data from the Global Adult Tobacco Survey (GATS).

[35] Lopez A.D., Collishaw N.E., Piha T. (1994). A descriptive model of the cigarette epidemic in developed countries. Tob. Control.

[36] Pan Z. (2004). Socioeconomic predictors of smoking and smoking frequency in urban China: Evidence of smoking as a social function. Health Promot. Int..

[37] Perlman F., Bobak M., Gilmore A., McKee M. (2007). Trends in the prevalence of smoking in Russia during the transition to a market economy. Tob. Control.

[38] Shang C., Huang J., Cheng K.W., Li Q., Chaloupka F.J. (2016). Global Evidence on the association between POS advertising bans and youth smoking participation. Int. J. Environ. Res. Public Health.

[39] Shang C., Huang J., Li Q., Chaloupka F.J. (2015). The association between Point-of-Sale Advertising Bans and Youth Experimental Smoking: Findings from the Global Youth Tobacco Survey (GYTS). AIMS Public Health.

[40] Angrist J.D., Pischke J.S. (2008). Chapter 8: Nonstandard Standard Error Issues. Mostly Harmless Econometrics: An Empiricist’s Companion.

